# Supplementary material for: Complete structure of the bacterial flagellar hook reveals extensive set of stabilizing interactions
Source: Nat Commun. 2016 Nov 4;7:13425. doi: 10.1038/ncomms13425 (PMC5097172; doi:10.1038/ncomms13425)
Supplement: Supplementary Information — Supplementary Figures 1-7, Supplementary Tables 1-4 and Supplementary References. [file ncomms13425-s1.pdf]

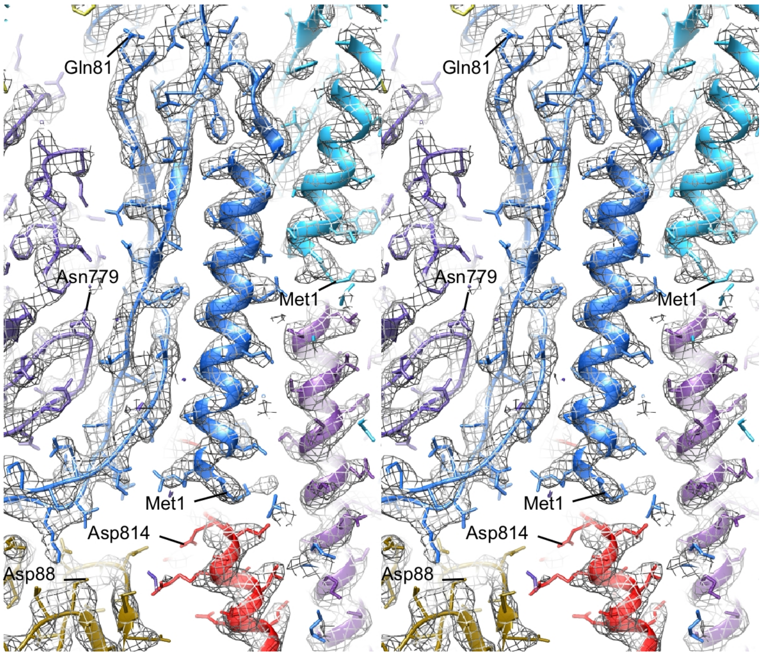

Supplementary Figure 1: Stereo view of cryo-EM density map (contoured at 2 sigma) superimposed with part of the structure of FlgEcj showing segments of the L-stretch and terminal α-helices in domain D0. The blue and cyan α-helices are those of the lower-end of N-terminal chains. The red and purple α-helices are the top-end of C-terminal chains. Each chain is represented by a different colour.

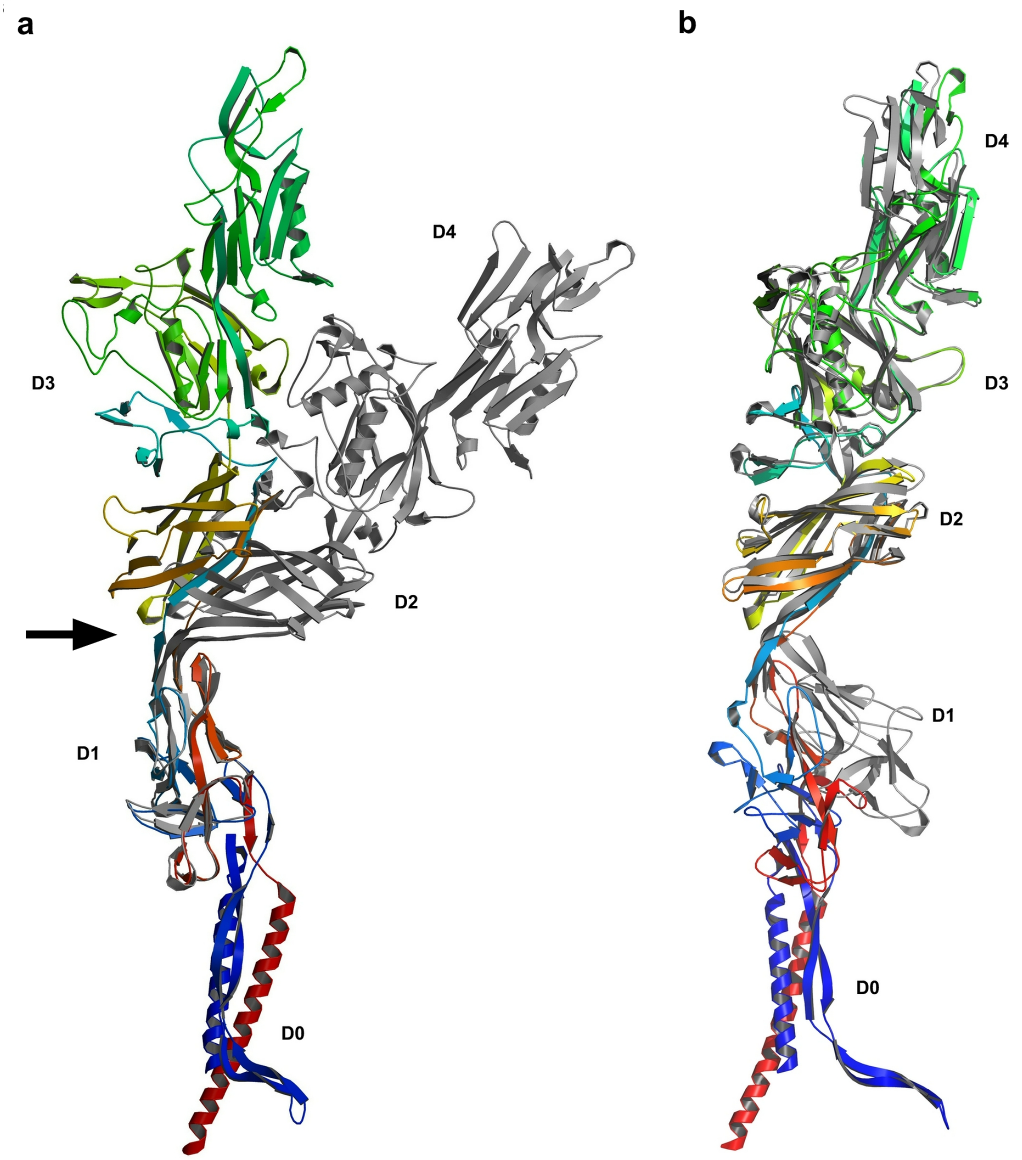

Supplementary Figure 2: **Comparison of the structure obtained by X-ray crystallography and by cryo-electron microscopy**. Domain orientations for FlgEcj in the hook (rainbow colours) as determined by cryo-EM, and in the crystal (grey). The hinge region between domains D1 and D2 is marked by a black arrow. Domains D1 aligned with a root mean square deviation (RMSD) of 0.93 Å (**a**). Domains D2 to D4 aligned with an RMSD of 1.35 Å (**b**). Figure prepared with MOLSCRIPT[1] and RASTER3D[2].

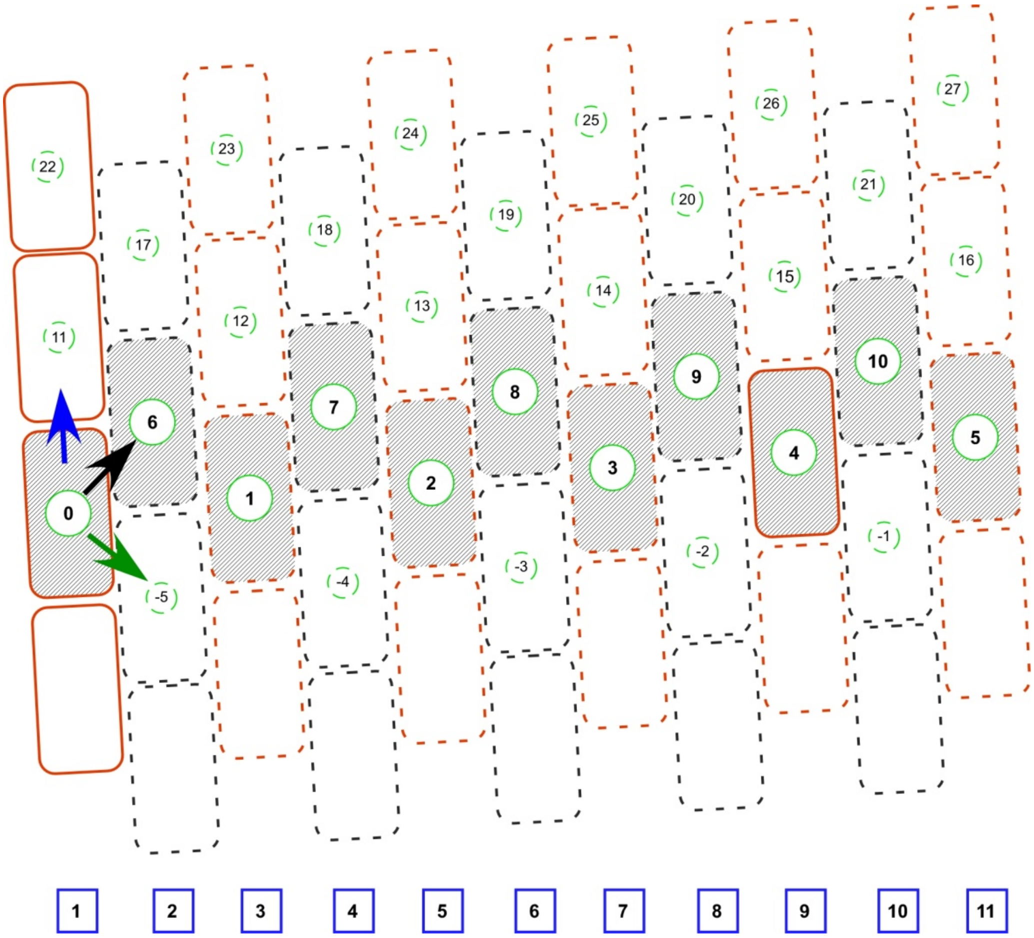

Supplementary Figure 3: **Schematic representation of the hook assembly**. The hook is a helical structure that is characterized by rotation and rise along its axis. It can be described as a helical assembly of a single protein, FlgE, with rise of 4.185 Å and rotation of 64.34º. The different subunits are packed to make eleven protofilaments. Thus the hook can also be described as a packing of eleven protofilaments (blue squared numbers). Each box represents a molecule of FlgE. The circled indices are the sequential numbers of molecules in the order of their appearance. These circled numbers will also define the direction of interactions among the molecules that make the assembly. The arrows represent some of the interactions found in the hook in the “6-start” (black), in the “11-start” (blue) and in the “minus 5-start” (green) helical directions.

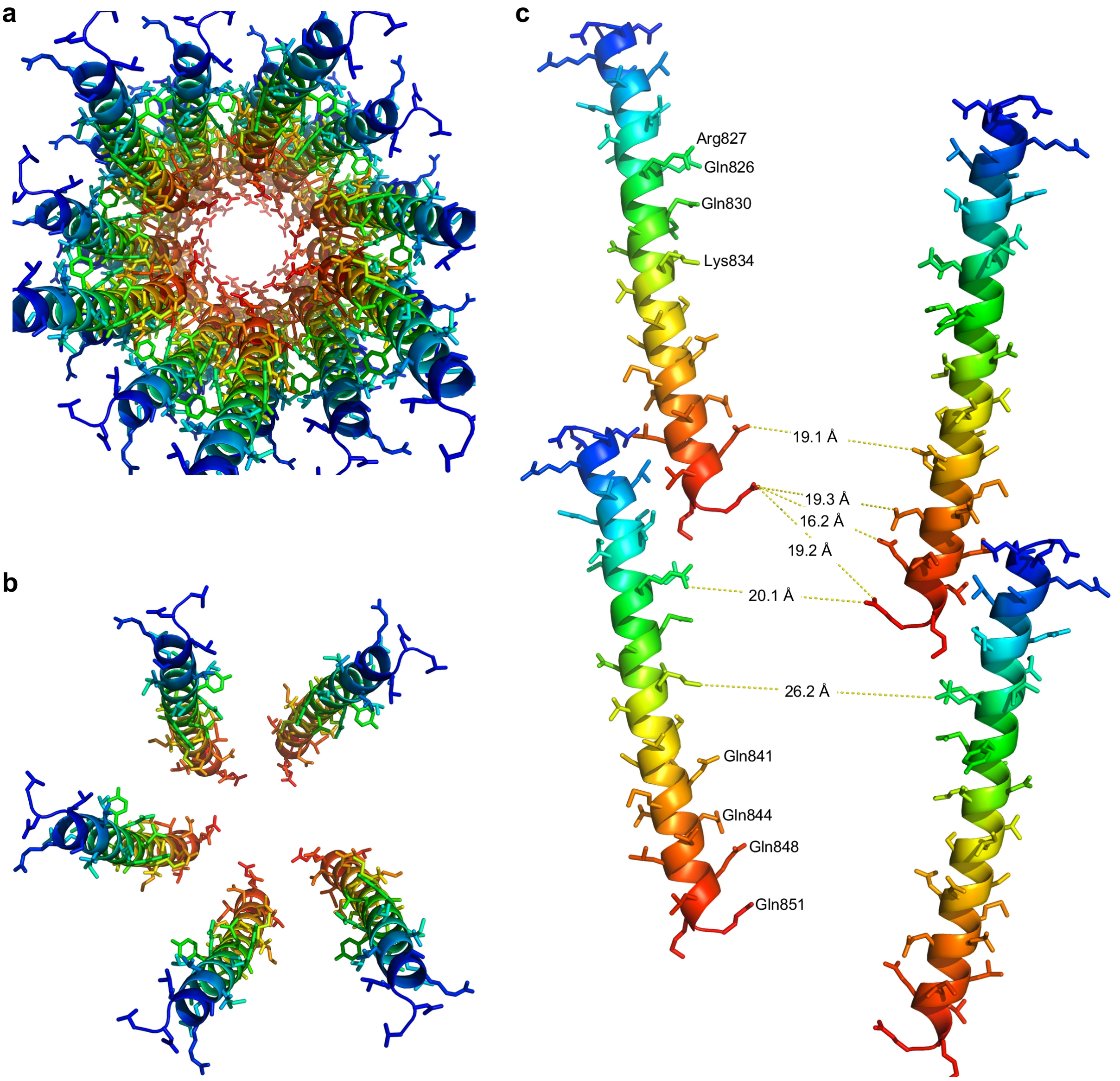

Supplementary Figure 4: **The central channel in the hook**. View in projection from the distal end of the central channel represented by the C-termini of 22 consecutive molecules (**a**) and of five consecutive molecules (**b**). Lateral view of the channel represented by C-termini taken from molecules on opposite side of the channel (**c**). The distances show the variations of the channel diameter. Figure prepared with PyMOL[3].

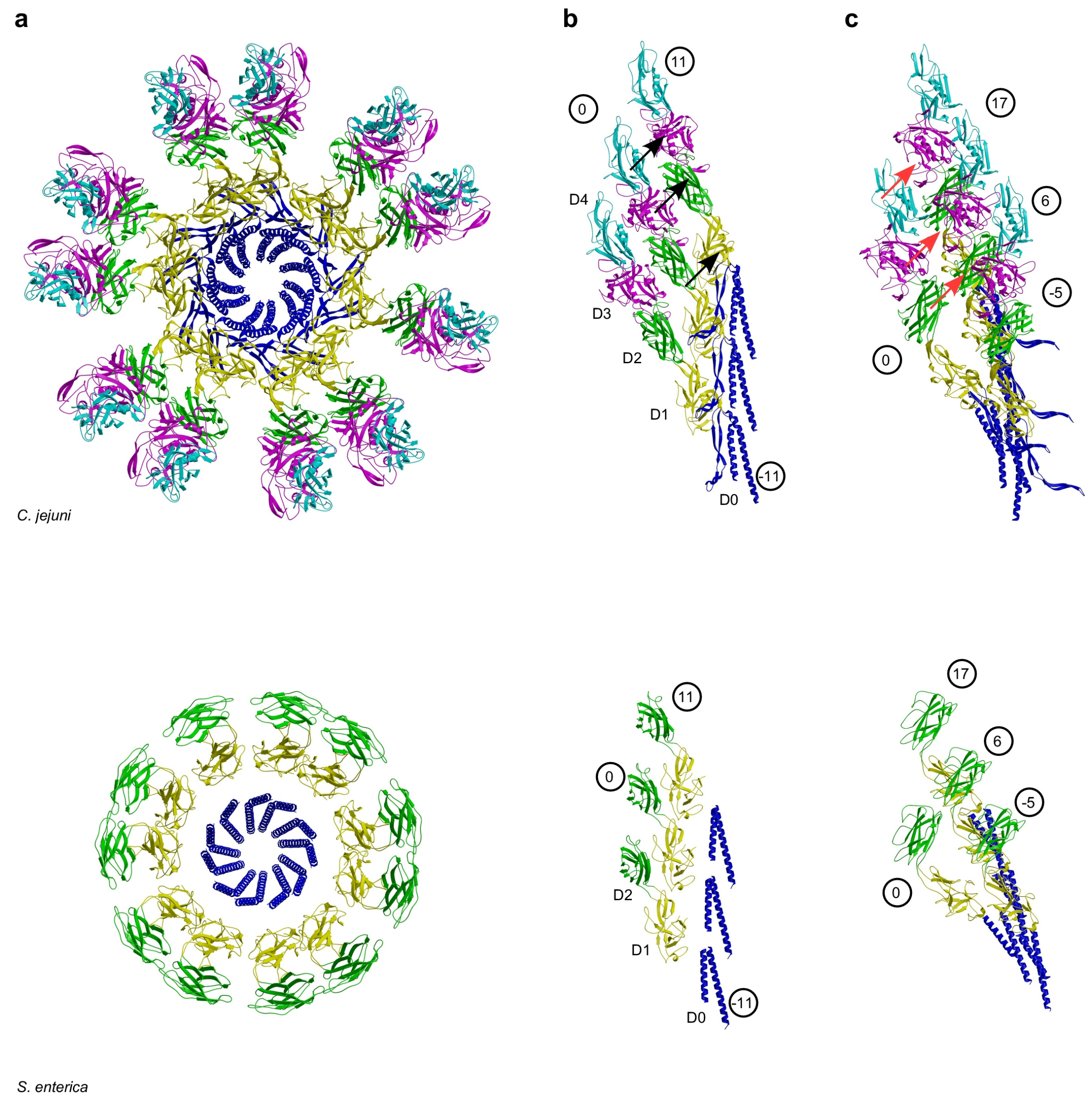

Supplementary Figure 5: **Hook structures from *C. jejuni* and from *S. enterica*.** Comparison of the hook structures of *C. jejuni* (upper cell) and of *S. enterica* (lower cell). Two-turn of the hook showing a ring made of 11 molecules in *C. jejuni* and in *S. enterica* (**a**). Interactions within a protofilament (**b**) and between protofilaments (**c**). Domain D0 is in blue, D1 in yellow, D2 in green, D3 in magenta and D4 in cyan. The circled numbers indicate the order of appearance of FlgE molecules during the building of the hook. Figure was prepared with MOLSCRIPT[1] and RASTER3D[2].

a

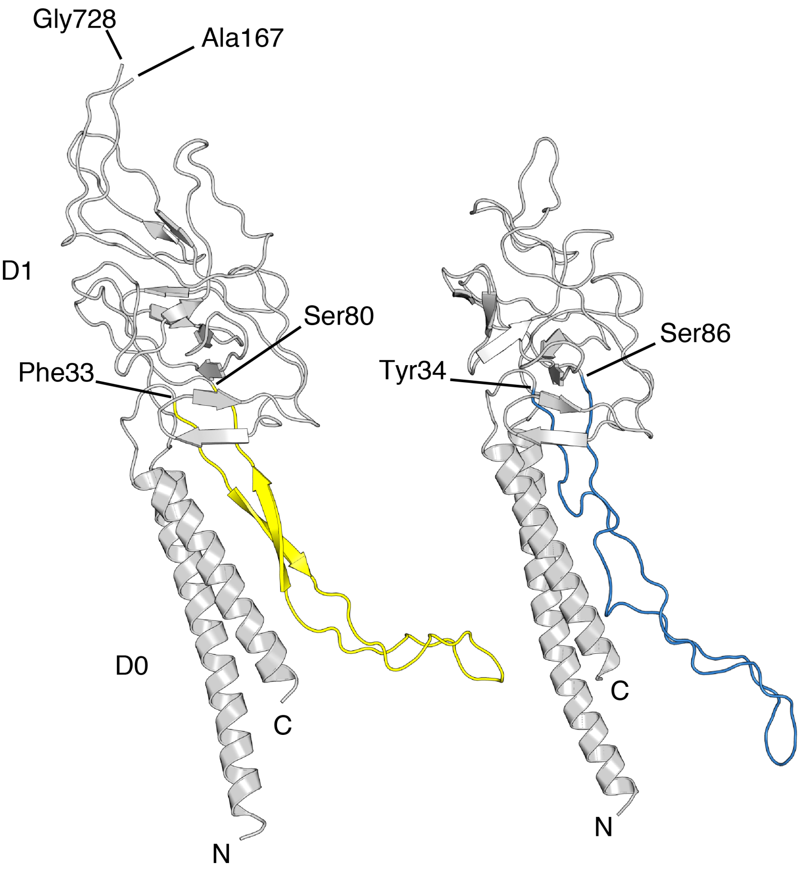

b

```
            33                                                   80
FLGE        FKYSR---ADFGTMFSQTVKIATAPTDGRGGSNPLQIGL--GVSVSSTTRIHS
            :|      |||  :| :|          |  |  ::  :     ||      |
FLGG        YKRDDVVIADFKRIFKETQDELPIENHTRDASRFVNTTIDGIPQVSQEYTDFS
            34                                                   86
```

Supplementary Figure 6: Structural comparison of the L-stretch regions of FlgE and FlgG from *C. jejuni*. (**a**) A homology model of FlgG (right) was build with MODELLER[4] using the D0 and D1 domains of FlgE (left) as a template. The L-stretch regions encompassing residues from Phe33 to Ser80 of FlgE and from Tyr34 to Ser86 of FlgG are coloured in yellow and in blue, respectively. Figure was prepared with PyMOL[3]. (**b**) A pairwise sequence alignment of the regions was calculated with EMBOSS Needle (www.ebi.ac.uk/Tools/psa/emboss_needle). Conserved and similar residues are shown as vertical line “|” and colon “:”, respectively. Sequence gaps are shown as hyphen “-”.

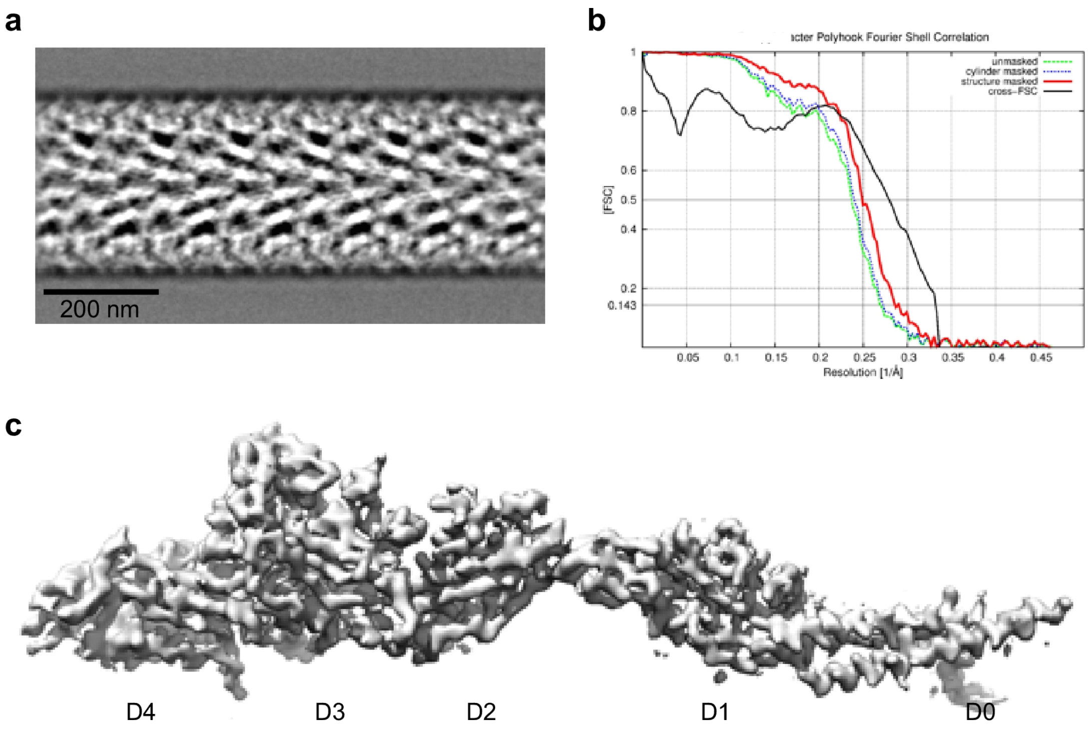

Supplementary Figure 7: **Cryo-electron microscopy data.** (**a**) Representative class average image (one of 20 classes) from approximately 4500 segmented projections. This image was used to determine the initial helical parameter using a real space symmetry exploration procedure implemented in the program SPRING[5]. (**b**) Fourier shell correlation between two half sets of independently refined datasets ("gold standard FSC", reconstruction using signal-to-noise weighting[5,6]. Resolution of the map is indicated as 3.5 Å (FSC=0.14) when the structure is masked with a soft mask (red curve). The resolution of the cylindrically masked and unmasked structure is measured as 3.7 Å (FSC=0.14, blue and green curves). The black curve is a cross-FSC between an excised segment containing one asymmetric unit and density generated from the fitted model (structure factors calculated with SFALL[7] to 3.0 Å and map created with FFT[7]. The cross-FSC indicates a resolution of 3.5 Å (FSC=0.5), which is consistent with the resolution estimate between half-sets (FSC=0.14) of the soft masked structure (red curve). (**c**) Iso-contour surface of one asymmetric unit excised from the reconstructed electron potential contoured at 1.8 sigma (rendered with UCSF Chimera[8]).

Supplementary Table 1: Sequence alignment of the C-terminal helix of FlgE from *C. jejuni* strains 81116 and 11168, from *S. enterica* and *C. crescentus*. Residues protruding into the channel are in purple. Residues lining the channel are in green. The " * " represents the conserved residues and the " **:** " represent residues that are replaced with a similar amino acid residue.

```
CJ_81116         VDLSRSLTELIIIQRGYQANSKTISTSDQMLQTLIQLKQ 852
CJ_11168         VDLSRSLTELIIIQRGYQANSKTISTSDQMLQTLIQLKQ 865
S. enterica      VDLSKELVNMIVAQRNYQSNAQTIKTQDQILNTLVNLR- 403
C. crescentus    VDLSQEFTGLITTQRAYSASSKIITTADQMLEELLNIKR 591
                 ****: :  :*  ** * : :: * * **:*: *::::
```

Supplementary Table 2: Strains of bacteria and plasmids.

Strain or plasmid	Genotype or description	Reference or source
Escherichia coli		
NEB 5-alpha	K-12 strain derivative	New England Biolabs, USA
Campylobacter jejuni		
C. jejuni 81116	Genetically stable, motile strain	9
C. jejuni 81116 *fliK*::Cam^R	*C. jejuni* 81116 *fliK*::Cam^R mutant strain derivative. Produces flagellar polyhooks	This study
CB991	*C. jejuni* 81116 Δ*flgE*::Km^R	This study
CB-A9	*C. jejuni* 81116 Δ*flgE*::Km^R 16S rRNA-*flgE*-Apr^R-tRNAala	This study
CB-A46	*C. jejuni* 81116 Δ*flgE*::Km^R 16S rRNA-*flgE*(*1-236*::*604-852*)-Apr^R-tRNAala	This study
**Plasmid**		
pUC19	High copy number plasmid in *E. coli*; Amp^R	Thermo Fisher Scientific, USA
pJMK30	pUC19 carrying *Campylobacter coli* Km^R cassette	10
pRRA	Vector for genome integration at *C. jejuni* NCTC 11168 rRNA loci with Apr^R selection	11
pCB950	pUC19 carrying *C. jejuni* 81116 16S rRNA-tRNAala-tRNAile-28S rRNA genes	This study
pCB951	pUC19 carrying *C. jejuni* 81116 *flgE* gene region	This study
pCB952	Vector for genome integration at *C. jejuni* 81116 rRNA loci with Apr^R selection	This study
pCB956	pUC19 carrying 16S rRNA-*flgE*-Apr^R-tRNAala-tRNAile-28S rRNA	This study
pCB963	pUC19 carrying Δ*flgE*::Km^R; *flgE* gene region with the *flgE* gene exchanged with a kanamycin-resistance gene	This study
pCB966	pUC19 carrying 16S rRNA-*flgE*(*1-236*::*604-852*)-Apr^R-tRNAala-tRNAile-28S rRNA	This study

Supplementary Table 3: Oligonucleotide primers.

Name	Sequence (5` to 3') *
**Primers used to make plasmid pCB950**	
Fd-pUC19-81116-RNA	gaccatgattacgccaagctt**CTGGAACTCAACTGACGCTAAG**
Rv-pUC19-81116-RNA	aattcgagctcggtacc**CTCTTGCACATTGCAGTCCTAC**
**Primers used to make plasmid pCB951**	
Fd-pUC19-CamflgEr	gaccatgattacgccaagctt**GGTATAGATCCTGGTTCAAGAAATTGCGG**
Rv-pUC19-CamflgEr	aattcgagctcggtacc**GGCACTACCATATCGCTACAACATTC**
**Primers used to make plasmid pCB952**	
Fd-pCB950-Linear	**CTAGAGTACAAAGTAATAAGTCTCACAACTATTACTTC**
Rv-pCB950-Linear	**CTAGAAAGGAGGTGATCCAACCG**
Fd-Apr-cassette	gatcacctcctttctag**TCGAGGTCGACGGTATCG**
Rv-Apr-cassette	agacttattactttgtactctag**GGCCGCTCTAGAGGATCC**
**Primers used to make plasmid pCB956**	
Fd-pCB952-Linear	**TCGAGGTCGACGGTATCG**
Rv-pCB952-Linear	**CTAGAAAGGAGGTGATCCAACCG**
Fd-flgE-Int	gatcacctcctttctag**TTTTAGTAATTTTTATTACACAAAAGCCCAAAGCTTCATAAAC**
Rv-flgE-Int	taccgtcgacctcga**ATTTTATAGAATACTAAGCTTAAATTATTGTTTAAGCTGGATTAGAGTTTG**
**Primers used to make plasmid pCB963**	
Fd-pCB951-Linear	**CAAAGAGGTTATCAAGCAAAC**
Rv-pCB951-Linear	**CTTAAATCCTTTATAAAATATTGCTTTAAAAAATTTAAC**
Fd-flgE-Km-swap	atattttataaaggatttaag**ATGGCTAAAATGAGAATATCAC**
Rv-flgE-Km-swap	gcttgataacctctttg**CTAAAACAATTCATCCAGTAAAATATAATATTTTATTTTCTCCCAATC**
**Primers used to make plasmid pCB966**	
Fd-CamflgE(1810-1832)	**CAACTTAAGCTTTCTGCTTTTTC**
Rv-CamflgE(691-708)	**AGCATCTACACCCTTTTC**

* The bases highlighted in bold hybridise to the template in PCR amplification or site-directed mutagenesis reactions.

Supplementary Table 4: Refinement and model statistics.

Data collection	
Particles	70,477 segments at 23-Å intervals
Pixel size (Å)	1.08
Defocus range (µm)	-0.5 ~ -2.5
Voltage (kV)	300.0
Detector	Falcon II
Dose rate	50 electrons/pixel/sec
Dose fractionation	18 frames/sec (2.4 electrons/$Å^2$/sec)
Total electron dose (e/$Å^2$)	12 (frames 1-5)
Model composition	
Non-hydrogen atoms	6363
Protein residues	851
Refinement	
Resolution (Å)	3.5
Map sharpening B-factor ($Å^2$)	-100
Fourier Shell Correlation	0.14
Helical symmetry (rise, rotation)	4.185 Å, 64.34 degrees
Rms deviations	
Bonds (Å)	0.0079
Angles (°)	1.13
Validation (protein)	
Molprobity score	
Clashscore, all atoms	15.78
Good rotamers (%)	100
Ramachandran plot	
Favored (%)	88.93
Allowed (%)	8.83
Outliers (%)	2.24

Supplementary References:

1. Kraulis, P. J. MOLSCRIPT: a program to produce both detailed and schematic plots of protein structures. *J. Appl. Crystal.* **24**, 946-950 (1991).

2. Merritt, E. A. & Bacon, D. J. Raster3D: Photorealistic molecular graphics. *Methods Enzymol.* **277**, 505-524 (1997).

3. DeLano, W. L. The PyMOL Molecular Graphics System (www.pymol.org) (2002).

4. Webb, B. & Sali, A. Comparative Protein Structure Modeling Using MODELLER. *Curr. Protoc. Bioinform.* **54**, 5.6.1-5.6.37 (2014).

5. Desfosses, A., Ciuffa, R., Gutsche, I. & Sachse, C. SPRING - an image processing package for single-particle based helical reconstruction from electron cryomicrographs. *J. Struct. Biol.* **185**, 15-26 (2014).

6. Rosenthal, P. B. & Henderson, R. Optimal determination of particle orientation, absolute hand, and contrast loss in single-particle electron cryomicroscopy. *J. Mol. Biol.* **333**, 721-745 (2003).

7. Winn, M. D. *et al*. Overview of the CCP4 suite and current developments. *Acta Crystallogr. D Biol. Crystallogr.* **67**, 235-242 (2011).

8. Pettersen, E. F. *et al*. UCSF Chimera: A visualization system for exploratory research and analysis. *J. Comput. Chem.* **25**, 1605-1612 (2004).

9. Pearson, B. M. *et al*. The complete genome sequence of *Campylobacter jejuni* strain 81116 (NCTC11828). *J. Bacteriol.* **189**, 8402-8403 (2007).

10. van Vliet, A. H., Wooldridge, K. G. & Ketley, J. M. Iron-responsive gene regulation in a *Campylobacter jejuni fur* mutant. *J. Bacteriol*. **180**, 5291-5298 (1998).

11. Cameron, A. & Gaynor, E. C. Hygromycin B and apramycin antibiotic resistance cassettes for use in *Campylobacter jejuni*. *PLoS ONE* **9**, e95084 (2014).
